# Anomalous Right Subclavian Artery-Esophageal Fistulae

**DOI:** 10.1155/2018/7541904

**Published:** 2018-03-01

**Authors:** Courtney Brooke Shires, Michael J. Rohrer

**Affiliations:** ^1^Department of Otolaryngology, Head and Neck Surgery, University of Tennessee Health Science Center, 910 Madison Ave., Suite 430, Memphis, TN 38163, USA; ^2^Department of Surgery, Division of Vascular and Endovascular Surgery, University of Tennessee Health Science Center, 910 Madison Ave., Second Floor, Memphis, TN 38163, USA

## Abstract

An aberrant right subclavian artery (ARSA) is the most common aortic arch anomaly, but only 19 previous cases of ARSA-esophageal fistula have been reported. Six patients have survived their bleeding episode. We describe the case of a 44-year-old woman who developed massive hemoptysis. Laryngoscopy, bronchoscopy, head and neck angiogram, and median sternotomy did not reveal what was presumed initially to be a tracheoinnominate fistula. Contrasted CT showed an anomalous subclavian artery posterior to the esophagus. Given the technical challenge of approaches for this pathology, the patient was unfit for open surgical repair. Therefore, endovascular covered stent grafts were deployed spanning the segment of the subclavian artery in continuity with the esophagus, via a right brachial artery approach. Unfortunately, the patient died after successful placement of the grafts.

## 1. Introduction

Artery-esophageal fistulae are rare but can cause massive and life-threatening hemorrhage. Prompt diagnosis and treatment are mandatory if the patient is to be saved, since these fistulae are fatal in a majority of cases. These fistulae most often develop secondary to prior thoracic operations, infection, neoplasm, foreign body, or radiotherapy and typically involve a fistula between the esophagus and the adjacent descending thoracic aorta [1–3]. Fistulae between the subclavian artery and esophagus are extremely rare and anatomically possible in the presence of an anomalous right subclavian artery which arises from the proximal descending thoracic aorta and courses behind the esophagus to supply the right arm.

## 2. Case Report

A 44-year-old woman with a history of gastroesophageal reflux disease and hypertension presented with worsening shortness of breath. She was found to have pneumonia and required intubation. She developed multiple complications, including sepsis, adult respiratory distress syndrome, acute renal failure, intensive care neuropathy, cardiac arrhythmias, and cardiac arrest. Approximately three weeks after hospitalization, a tracheostomy tube was placed. She then developed intra-abdominal abscesses after a gastrostomy tube became dislodged. She underwent exploratory laparotomy with abscess drainage and closure of the gastrostomy and was maintained on total parenteral nutrition.

Four months after admission to the hospital, a nasogastric tube was placed and enteral nutrition was started. Sixteen days later, she experienced an episode of substantial bleeding, and it was not clear whether this came from the airway or oropharynx. Her hematocrit was 20 percent, platelets were 396,000, and blood urea nitrogen was 13 mg/dL. She was resuscitated with packed red blood cells, platelets, cryoprecipitate, and fresh frozen plasma; selective carotid arteriography of the head and neck, direct laryngoscopy, and bronchoscopy were unremarkable.

**Figure 1:**
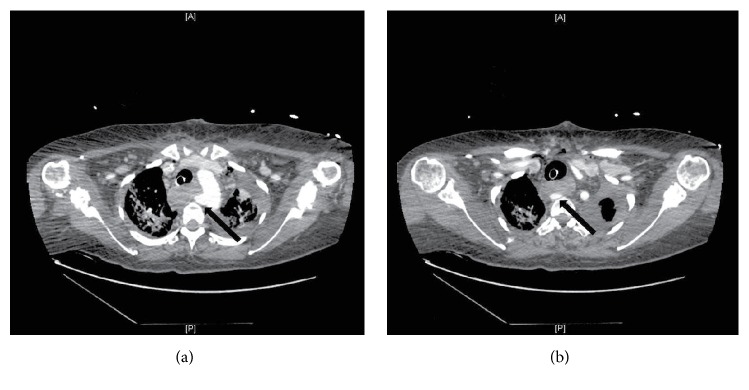
Sequential CT scan images show origin of right subclavian artery from the proximal descending thoracic aorta ((a), black arrow) and coursing posterior to the esophagus ((b), black arrow).

**Figure 2:**
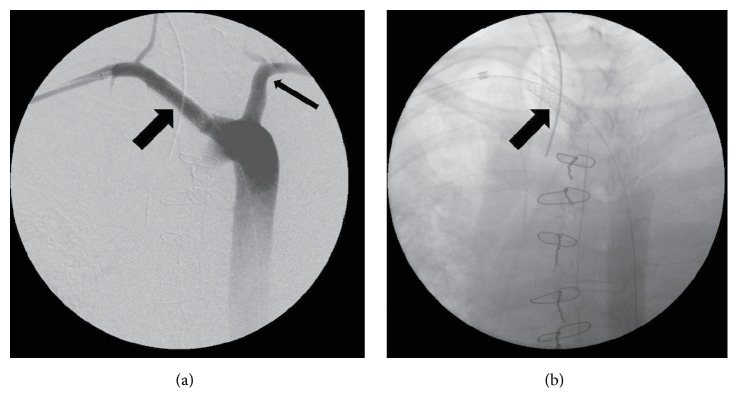
(a) Intraoperative angiogram performed in an AP projection through a sheath positioned in the right axillary artery from a right brachial approach. The right subclavian artery is seen (wide arrow) as is the left subclavian artery (narrow arrow). (b) Intraoperative image in the same projection after deployment of a balloon expandable covered stent at level of esophageal-arterial communication. The arrow indicates the position of the covered stent.

Three days later, she again experienced severe bleeding, which was initially felt to have originated from her airway. She was taken to the operating room by the vascular surgery team with the preoperative impression that she had a tracheoinnominate fistula. Median sternotomy revealed a right carotid artery arising directly from the aortic arch with no innominate or right subclavian artery in the anterior mediastinum. No artery to trachea fistula was identified and a provisional diagnosis of aberrant right subclavian artery with subclavian to esophageal fistula was made given the observation of the absence of the subclavian and innominate arteries in the usual location. The sternotomy was closed and a CT of the chest was performed, which demonstrated the anomalous right subclavian artery without aneurysmal degeneration (Figures 1(a) and 1(b)). She was taken back to the operating room for endovascular covered stent grafting of the segment of the subclavian artery in continuity with the esophagus. An angiogram was performed (Figure 2(a)) and three covered stents were deployed to span the artery-esophageal fistula via a right brachial artery approach (Figure 2(b)). Successful control of the bleeding was accomplished, but she experienced a cardiopulmonary arrest and died despite resuscitative efforts.

## 3. Discussion

The presence of an aberrant right subclavian artery (ARSA) is the most common aortic arch anomaly, present in 0.2–2.5% of the population [4, 5]. The discovery of this variation is usually an incidental finding on imaging studies performed for other reasons, but the anomaly is more prevalent in patients with Down's syndrome and patients with chromosome 22q11 deletions [5–8]. Previous reports have suggested that this anomaly is more common in females (65–72%), but in another study it had equal sex prevalence [6]. When an anomalous subclavian artery is present, it courses posterior to the esophagus in 80% of cases, between the esophagus and trachea in 15%, and anterior to the trachea in 5% [9, 10]. This anomalous artery often becomes aneurysmal, resulting in the development of what is known as “Kommerell's diverticulum.”

Despite the relative frequency of ARSA, our case of ARSA-esophageal fistula is only the twentieth reported case of ARSA-esophageal fistula, which has involved both aneurysmal and nonaneurysmal anomalous arteries. None of the patients who developed an ARSA-esophageal fistula associated with an aneurysmal artery have survived, and none of the first ten reported cases of nonaneurysmal ARSA-esophageal fistula survived their bleeding episode. Hemorrhage was fatal in fourteen of these twenty patients, bringing the mortality rate in reported cases to 70%.

Diagnosis of the ARSA-esophageal fistula is challenging. In most cases, esophagoscopy cannot be used to assist in diagnosis of acute hemorrhage because of the extreme volume of hematemesis [3]. Upper endoscopy has been used in six reported cases and was able to identify the source of bleeding in only three patients [11–13]. This is comparable to the sensitivity of EGD in diagnosing artery-esophageal fistulas of 38% [11]. The risk of EGD dislodging a hemostatic clot and inciting recurrent hemorrhage has been recognized, and McFaddin et al. advocated EGD in the operating room with preparation for thoracotomy should this become necessary [3, 14]. In stable patients, cervicothoracic CT scanning with contrast for visualization of ARSA is the modality of choice. CT scanning can provide strong support of the diagnosis of artery-esophageal fistula [3]. Angiography can be performed in the operating room and can definitively define the presence of the anomalous subclavian artery and therefore heighten the index of suspicion for the presence of an anomalous subclavian to esophageal fistula.

Management of patients with bleeding from an anomalous subclavian artery to esophagus fistula is very challenging since bleeding in these patients presents as abrupt, rapidly exsanguinating hematemesis, and the etiology is from an unusual and unexpected source. Sentinel bleeding was reported in four of the twenty patients with ARSA-esophageal fistula, including our patient. In most patients with nonaneurysmal ARSA-esophageal fistula, presentation is abrupt, massive arterial bleeding for several days to weeks after placement of a nasogastric or endotracheal tube [3]. The occurrence of an initially limited episode of bleeding provides an opportunity to make an anatomic diagnosis and treat the problem before the final exsanguinating event.

Initial management can involve the use of a Sengstaken-Blakemore tube, which can be used to temporize the bleeding while the patient is stabilized, as in patients with any artery-esophageal fistula. In actively bleeding patients, right thoracotomy is recommended by most physicians to treat the problem. This results in appropriate exposure of both the anomalous artery and esophagus. In general, infants do not require revascularization of the right arm, because of excellent collateral circulation [2]. Adults, however, typically require revascularization after ligation and division of the aberrant vessel to prevent subclavian steal syndrome [13, 15]. Primary esophageal repair should be performed if limited mediastinal contamination has occurred [2]. Muscle and fascial flaps are useful to provide a barrier between the esophagus and the involved artery.

In patients who are at high surgical risk and not likely to survive a thoracotomy, or those individuals with a limited life expectancy, arteriography with endovascular stent grafting may be used. Inman et al. advocated placing stents to span the width of the posterior esophagus overlapping anomalous artery on both the distal and proximal ends [4]. Although stent graft placement in a contaminated wound is not recommended as definitive therapy, it may be used as a temporizing measure [16, 17]. However, in 2008, Magagna et al. successfully placed an endovascular prosthesis in a ARSA-esophageal fistula of a 73-year-old woman undergoing chemoradiation therapy after total laryngectomy for laryngeal cancer who made a complete and prolonged recovery [18]. We were able to deploy three stents in the right subclavian artery in our patient. Unfortunately, she experienced a cardiac arrest in spite of successful deployment of covered stents and did not survive.

Associated risk factors for the development of ARSA-esophageal fistulization have varied (Table 1). Three patients had previously undergone grafting of the anomalous artery. Four patients were advanced in age and were found to have aneurysms of the ARSA. Three patients, including our patient, had been receiving long term corticosteroids. A child with Down's syndrome experienced fistulization from an esophageal stent placed after caustic ingestion. The most common associated risk factor, however, has been the prolonged presence of a nasogastric tube, which has been the case in thirteen of the nineteen patients reported in the literature. It is evident that prolonged placement of nasogastric or endotracheal tube or vascular grafts may result in significant compression, pressure, or friction with the ARSA, which is adjacent to the esophagus, resulting in formation of a fistula [3].

It is, therefore, logical to recommend avoiding prolonged nasogastric or endotracheal intubation in patients with known aberrant right subclavian artery. Feugier et al. have suggested screening of intensive care patients before long term placement of nasogastric tubes [3]. Transesophageal ultrasound is usually diagnostic, but this diagnosis can often be made as an incidental finding on a chest CT scan ordered for other reasons. The only chest CT that this patient had undergone was obtained earlier in her care when there was concern about contrast-related nephrotoxicity and therefore no intravenous contrast was given. At the time, she had severe lung consolidation and the anomalous artery was not recognized, even in retrospect.

Nasogastric tubes should be removed and gastrostomy performed in patients with ARSA on imaging [3]. Our patient underwent prolonged nasogastric tube placement for nutritive support secondary to dislodged PEG tube with resulting intraabdominal abscesses. Had we known she had an anomalous artery, perhaps reoperative surgical gastrostomy, jejunostomy placement, or the use of TPN would have been appropriate alternative sources of nutrition.

## 4. Conclusion

The course of an anomalous subclavian artery usually brings it posterior and contiguous with the esophagus. Although usually asymptomatic, the presence of prolonged nasogastric intubation may predispose an erosion of the esophagus into the adjacent artery leading to an arterial-esophageal fistula. Subclavian esophageal fistulae do not always present with sentinel bleeding. In stable patients, CT with contrast, angiography, and endoscopy may be useful in diagnosis and may reveal an otherwise unsuspected source. Once a diagnosis is made, bleeding from an ARSA-esophageal fistula may be temporized with a Sengstaken-Blakemore tube. Open surgical repair remains the gold standard of treatment, although endovascular stent grafts may be important to control life-threatening bleeding. The presence of a nasogastric tube is associated with development of these fistulae; therefore, prolonged NG tube placement should be carefully considered in patients with known anomalous right subclavian arteries.

## Figures and Tables

**Table 1:** Reported cases of anomalous subclavian artery-esophageal fistulae.

Study	Age (years)	Sex	Pathology	Cause	Predisposing factors	Time lag after intubation (days)	Outcome	Treatment	Sentinel bleed
Lynn, 1969	57	M		Aneurysm	Aneurysm		Fatal		
Reynes, 1976	72	F		Aneurysm	Aneurysm		Fatal		Yes
Merchant et al., 1977	17	F	Recovery after cesarian section	NGT		9	Fatal		
Livesay et al., 1982	25	M	Head trauma	ETT/NGT		13	Fatal		
Jungck and Puschel, 1983	6	M	Multiple trauma	ETT/NGT		42	Fatal		
Belkin et al., 1984	27	M	ENT cancer	NGT		60	Fatal		
Edwards et al., 1984	36	F	Subarachnoid hemorrhage	NGT	Corticotherapy	27	Fatal		Yes
Gosset et al., 1985	72	F	Recovery after aortic repair	ETT/NGT	Corticotherapy, infection, surgery	30	Fatal		
Guzzeta et al., 1989	0.4	F	Recovery after heart surgery	NGT			Survived		
Kulling, 1989		M			Aneurysm		Fatal		
Ikeda et al., 1991	9	M	Recovery after heart surgery	ETT/NGT	Surgery		Fatal		
Stone, 1990		M					Fatal		
Hirakata et al., 1991	55	M	Esophageal cancer	NGT	Surgery, irradiation, cancer	44	Survived		
Miller et al., 1996	11	F	Intracerebral hemorrhage	ETT/NGT		17	Survived	Surgery	No
Minyard and Smith, 2000	39	F	Head trauma	NGT			Fatal		
Feugier et al., 2003	24	M	Multiple trauma	ETT/NGT	Deceleration syndrome	31	Survived	Surgery	
Eynden, 2006	9	F		Esophageal stent	Caustic ingestion in Down syndrome patient with subsequent esophageal fissures and mediastinitis		Survived	Surgery	Yes
Inman et al., 2008	63	M		Salivary bypass tube	Chemotherapy and radiation, then salvage total laryngectomy and pharyngectomy		Fatal	Endovascular stent	Yes
Magagna et al., 2008	73	F			Laryngectomy, then chemotherapy and radiation therapy		Survived	Stent	Yes
Current study	41	F		NGT, tracheostomy tube	Corticotherapy, sepsis		Fatal	Stent	Yes
